# Better Neuronal Efficiency After Emotional Competences Training: An fMRI Study

**DOI:** 10.5334/pb.av

**Published:** 2014-07-21

**Authors:** Michel Hansenne, Delphine Nélis, Dorothée Feyers, Eric Salmon, Steve Majerus

**Affiliations:** 1 Department of Psychology, University of Liège, Belgium; 2 Cyclotron Research Centre, University of Liège, Belgium; 3 Belgian National Fund for Scientific Research (FNRS), Belgium

**Keywords:** emotional competencies, emotional regulation, training, prefrontal cortex, fMRI

## Abstract

Earlier studies demonstrated that adult emotional competences (EC) can be improved through relatively brief training. This increase has been investigated, thus far, using self-reported questionnaires and behavioral data. The aim of the present study was to evaluate the cerebral correlates underlying improvement in EC. An experimental group received an EC training and a control group received brief sessions of drama improvisation. Participants viewed negative, positive, and neutral pictures while attempting to decrease, increase, or not modulate their emotional reactions. Subjective reactions were assessed via on-line ratings. After the intervention, the training group showed less cerebral activity as compared to the control group within different regions related to emotional regulation and attention including prefrontal regions and the bilateral inferior parietal lobule, the right precentral gyrus and the intraparietal sulcus. These results suggest increased neural efficiency in the training group as a result of emotional competencies training.

## Introduction

As more and more evidence suggests, emotions do not only color people’s lives, but are absolutely essential to people’s survival and adaptation (Cosmides & Tooby, 2000). Emotions are central and useful in everyday life. Identify, express, understand, regulate, and use emotions are important to enhance well-being in general. Emotional competence (EC), also called emotional intelligence (EI) or emotional skills, includes five core competencies: identification, expression, understanding, regulation, and utilization of one’s emotions and those of others (Mayer & Salovey, 1997; Petrides & Furnham, 2003; Saarni, 1999).

A vast amount of research has documented a positive association between EC and well-being related variables (Van Rooy & Viswesvaran, 2004; Zeidner, Matthews, & Robert, 2009). Higher EC is linked to many positive outcomes, including greater well-being and higher self-esteem (Schutte, Malouff, Simunek, McKenley, & Hollander, 2002), better physical health (Austin, Saklofske, & Egan 2005; Luminet, de Timary, Buysschaert, & Luts, 2006; Suls, Wan, & Costa, 1995), better social relationships (Lopes et al., 2004; Lopes, Salovey, Côté, & Beers, 2005; Schutte et al., 2001), greater academic achievement (Leroy & Grégoire, 2007; Mavroveli & Sanchez-Ruiz, 2011; Petrides, Frederickson, & Furnham, 2004), reduced stress (Austin, Saklofske, & Mastoras, 2010), and higher job performance (Shahhosseini, Silong, Ismaill, & Uli, 2012).

Given these positive outcomes, it would be quite beneficial for people to strive to optimize their EC. There are many interventions designed to improve individual EC (Matthews, Zeidner, & Roberts, 2002). However, many fall short on some aspects. First, despite the huge expansion of EC development methods and the preliminary evidence for their effectiveness, especially with children (Zins, Weissberg, Wang, & Walberg, 2004), very few EC programs are based on a solid theoretical model and even fewer have been carefully tested (Matthews et al., 2002; Matthews, Zeidner, & Roberts, 2007). Second, these EC programs usually target only on some dimensions of EC (e.g., emotion identification but not emotion management) and add a number of skills that that lie outside the domain of emotional competencies, such as in the case of problem resolution, alcohol or drug prevention, and reduction of violence (e.g., Topping, Holmes, & Bremmer, 2000). Third, when evaluations of these programs exist, they are often limited to subjective impressions right after the training (given by teachers for EC training at school or by the director for EC training at work) without considering its long-term effects (Aber, Brown, & Henrich, 1999; Goleman, 1995; Matthews et al., 2002). Finally, few assessment of EC training to date has included a control group.

Therefore, in order to address these shortcomings, we developed an 18-hour EC intervention (Nélis, Quoidbach, Mikolajczak, & Hansenne, 2009). A similar procedure has also been developed by Di Fabio & Kenny (2011) with interesting results among high school students. Our EC intervention focuses on teaching theoretical knowledge about emotions (see Mikolajczak, Quoidbach, Kotsou, & Nélis, 2009 for a full description of the theoretical and empirical bases of the training). For example, Scherer’s (2001) model on the multiple components of emotion and Ekman’s (1971) work on facial expressions informed a large part of a module about the perception of emotion in oneself and in others. Likewise, effective emotion regulation strategies (e.g., Gross, 2007; Lazarus & Folkman, 1984) were used to develop a large part of the emotional regulation module. Our intervention focuses also on encouraging participants to apply specific emotional skills in their everyday life. Three studies have evaluated the validity of this training (Kotsou, Nélis, Grégoire, & Mikolajczak, 2011; Nélis, Quoidbach, Mikolajczak, & Hansenne, 2009; Nélis et al., 2011). Results showed that 18 hours of training with email follow-up were sufficient to significantly improve emotion regulation, emotion understanding, and overall EC. Moreover, long-term significant increases in extraversion and agreeableness as well as a decrease in neuroticism have been reported. Results also showed that the development of EC brought about positive changes in psychological well-being, subjective health, quality of social relationships, and employability. These effects were obtained using self-report questionnaires, objective (e.g., cortisol level) and informant-report measures.

In the present study, we wanted to explore the cerebral correlates underlying improvement in EC. Several lines of evidence suggest that brain activity can be modified after extended training. For instance, a strong line of research demonstrated that meditation is accompanied by changes in brain activity (Tomasino, Fregona, Skrap, & Fabbro, 2013). Davidson and colleagues (2003) reported an increase in left-sided anterior activation among participants after an 8-week course in mindfulness meditation-based stress reduction. Another study showed that expert meditators have less activation than novices in a network of brain regions typically involved in sustained attention including frontal and parietal regions, lateral occipital, insula, multiple thalamic nuclei, basal ganglia, and cerebellar regions, meaning that expert meditators exhibit greater neural efficiency (i.e., reduced functional activation) during relevant tasks (Brefczynski-Lewis, Lutz, Schaefer, Levinson, & Davidson, 2007). In the same vein, subjects with extensive musical training showed increased activation during music listening in the right superior and middle temporal gyri, the right inferior frontal gyrus, and the left supramarginal gyrus compared to non-musicians (Seung, Kyong, Woo, Lee, & Lee, 2005). Finally, neuroimaging findings indicate that psychotherapy can lead to regional brain metabolic changes in depression, obsessive-compulsive disorder, and spider phobia, mainly in frontal and amygdala regions (Baxter et al., 1992; Brody et al., 2001; Goldapple et al., 2004; Martin, Martin, Rai, Richardson, & Royall, 2001; Paquette et al., 2003; Schwartz, 1996; Straube, Glauer, Dilger, Mentzel, & Miltner, 2006).

The aim of the present functional magnetic resonance imaging (fMRI) study was to examine the changes in cerebral processing occurring after EC training. Since it was not possible to assess the five emotional competencies (e.g., identification, expression, understanding, utilization and regulation) in one task, we chose to examine only emotion regulation. Previous functional neuroimaging studies have depicted the brain regions involved in emotional regulation based on recent models suggesting two opposed but interacting processes elicited by emotionally stimuli: top-down control processes and automatic bottom-up processes (Ochsner, Silvers, & Buhle, 2012). In healthy individuals, regions of the prefrontal cortex (PFC), including the orbital frontal cortex and anterior cingulate cortex (ACC) were recruited during down-regulation of negative emotion (Kim & Hamann, 2007; Ochsner et al., 2004; Ochsner & Gross, 2005). The same regions were also recruited during up-regulation of negative emotion (van Reekum et al., 2007). Studies showed also that amygdala activation is modulated up or down depending on the regulatory goal (Kim & Hamann, 2007; Ochsner et al., 2004). In contrast, emotion dysregulation involves a lack of prefrontal down-regulation processes, leading to enhanced emotional reactivity revealed by higher activation within the amygdala. In addition, attentional orienting processes located in the ventral network (right temporal-parietal junction and the right ventral frontal cortex) is implicated in emotional regulation (Viviani, 2013; Vuilleumier, 2005).

Findings in the emotion regulation domain support an emerging multilevel functional architecture involved in cognitive emotion regulation (Ochsner & Gross, 2008). In this model, cognitive strategies modulate the activity of prefrontal and cingulate systems requested for attention, response selection, working memory, language, mental-state attribution, and autonomic control. Specifically, activated regions include dorsal portions of the prefrontal cortex implicated in working memory and selective attention, ventral portions of the prefrontal cortex that have been implicated in language or response inhibition, dorsal portions of the anterior cingulate cortex implicated in monitoring processes, and dorsal portions of the medial prefrontal cortex implicated in reflecting upon one’s own or someone else’s affective states. The regulatory effects of any given strategy can be understood in terms of that strategy’s reliance upon specific component control processes and the regulatory effects that those control processes exert on systems involved in various aspects of emotional responding, such as the amygdala which has been implicated in the detection and encoding of affectively arousing stimuli.

In the present study, two forms of emotional regulation will be examined: the down-regulation of negative emotions and the up-regulation of positive ones. These strategies are the two forms of regulation most often encountered in daily life (Gross, Richards, & John, 2006). Based on several studies, we predicted that the regulation processes would recruit top-down prefrontal and parietal regions generally implicated in cognitive and attentional control, and bottom-up emotion-processing regions such as the amygdala (Beauregard, Levesque, & Bourgouin, 2001; Kim & Hamann, 2007; Oschner et al., 2002; 2004). Emotion regulation would alter activity in the amygdala in line with the regulatory goal (Ochsner et al., 2002; 2004; Kim & Hamann, 2007). In addition, we postulated that the EC training should lead to a better control of emotion regulation, resulting in diminished cerebral activity in regions implicated in emotion regulation. More particularly, on the basis of previous studies suggesting that individuals characterized by higher developed skills tend to show greater neural efficiency (i.e., reduced functional activation) during relevant tasks (Haslinger et al., 2004; Killgore & Yurgelun-Todd, 2007; Olson et al., 2006), we hypothesized that the EC group would show less activation in prefrontal and parietal regions typically recruited in emotional regulation tasks.

## Method

### Participants

Thirty-six right-handed women participated in the experiment. The EC and the drama improvisation groups consisted of eighteen participants each with a mean age of 21 years (*SD* = 2.8 and 1.9, respectively). Participants in the EC program may have been involuntarily influenced by experimenter demand, expectations of improvement, and by group effects such as contact with a caring instructor and social support provided by the group. So, we formed a control group that took part in a drama improvisation training similar to the EC training in terms of the possibility to experience group dynamics and the opportunity to develop new relationships. Participants in both groups attended all the sessions and were blind to their scores throughout the study. They all gave their written informed consent to take part in the study, which was approved by the Ethics Committee of the Psychology School of the University of Liège. None of the participants had any history of neurological or psychiatric disorders. All participants were scanned before and after taking part in their respective program. Participants also completed a self-report measure of global emotional intelligence, the Trait Emotional Intelligence Questionnaire-Short Form (TEIQue-SF; Petrides & Furnham, 2006), which is comprised of thirty 7-point items providing a global measure of EC. This measure is a short version of the Trait Emotional Intelligence Questionnaire (TEIQue; Petrides & Furnham, 2003). The TEIQue shows excellent psychometric properties (see Mikolajczak, Luminet, Leroy, & Roy, 2007, for the psychometric properties of the French adaptation used in this study).

### EC intervention

The EC intervention consisted of either 3x6 hours (two days + one day two weeks after) or 6x3 hours (spread over 6 weeks). This interval between days or sessions aimed at allowing participants to apply their learning in their daily lives. Each session was designed to enhance a specific emotional competence: understanding emotions, identifying one’s own emotions, identifying others’ emotions, regulating one’s own emotions, regulating others’ emotions, and using positive emotions to foster well-being. The content of each session was based on short lectures, role-playing games, group discussions, and work in dyads. Participants were also provided with a personal diary in which they had to report daily one emotional experience. These emotional experiences had to be analyzed in light of the theory presented in class. Finally, various readings were also proposed. After the in-class training, an email-based follow-up was set up to optimize the transfer of knowledge into daily life. Participants received two emails per week for six weeks (12 emails in total). Each email included a theoretical reminder of the notions discussed in class and a practical exercise related to it. Emails were kept as short and simple as possible to increase the chances they were actually read and put into practice. The detailed outline of the sessions is presented in the Appendix.

### Drama improvisation intervention

The drama improvisation training consisted of 6x3-hours workshops. The workshops were prepared using a set of improvisational theatre manuals and taught by an improvisation practitioner. The outline of sessions was as follow: 1) warming up (i.e., relaxation, physical and vocal warm up, concentration, and stimulation of imagination), 2) basic exercises (i.e., acquisition of new precepts and tools), 3) group improvisation (i.e., integration of these new acquisitions), and 4) debriefing.

### Stimuli and task for fMRI acquisition

Participants were shown colored pictures that were designed to elicit either a negative (e.g., vermin, accidents, illness, domestic violence, pollution), positive (e.g., domestic pets, landscapes, babies, romantic couples), or neutral (e.g., domestic objective) affective responses. Two sets of 42 negative pictures, two sets of 42 positive pictures, and one set of 16 neutral pictures were selected from the International Affective Picture System (Lang, Bradley, & Cuthbert, 1995). Each set of negative and positive pictures was matched on normative ratings of arousal and valence (Lang et al., 1995), and was assigned to the three experimental conditions (i.e., increase, decrease, or watch condition). Neutral pictures were always assigned to the watch condition. In order to familiarize participants with the experimental procedure prior to the fMRI sessions, an additional set of 18 pictures was selected for a practice task.

The regulation task was the same as that used by Kim and Hamann (2007). Participants were instructed to either increase or decrease their emotional reactions to each picture. In the increase condition, participants were instructed to think about the positive pictures in such a way that they felt the emotions elicited by the pictures more intensely. In the decrease condition, participants were instructed to think about the negative pictures in such a way that they felt the emotions elicited by the pictures less intensely. In the watch condition, participants were instructed to view the picture in a natural way and not to try to change the emotion elicited by the picture. Before training, examples of regulation strategies were not given to participants.

### Procedure

**Figure 1:**
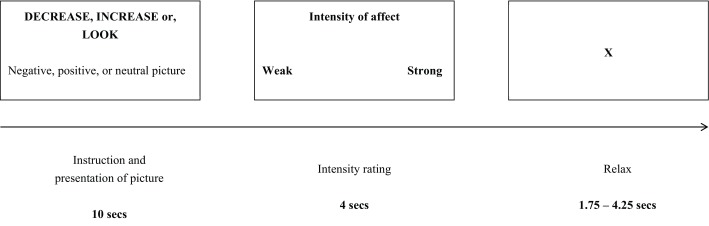
Design of the experimental trials. Timeline for events on each trial. An initial cue instructs participants to decrease, increase, or look. Below this instruction, the picture was presented. During the presentation of the picture, participants follow the instruction. Participants then provide a rating of their current affect and finally have a moment to relax before the onset of the next trial.

Participants were scanned before training and a second time eight weeks after the last group session. Prior to scanning, participants received instructions about the regulation task and performed a practice task with 18 pictures depicting similar contents to those presented during scanning. Once the practice trials were completed, the experimental task began. In the scanner, participants again completed the 18 practice trials to ensure that they were able to perform the task inside the scanner. In the task, a regulation instruction (increase, decrease, watch) was presented above the picture for 10 seconds. Next, a Likert scale ranging from 1 (weak) to 4 (strong) was presented for a duration of 5 seconds maximum, and participants were asked to rate the strength of the emotion they were currently feeling by pressing a button on an MRI-compatible response box. Just following the response to the rating scale, a fixation cross in a black screen was presented before the next trial for a random period comprised between 1.75 and 4.25 seconds while participants were instructed to rest (Figure 1). A total of 100 trials were completed. Twenty-one pictures were presented by condition (increase positive, decrease negative, watch positive and watch negative) and sixteen for the neutral condition (watch neutral).

### MRI acquisition

Data were acquired on a 3 Tesla scanner (Siemens, Allegra, Erlangen, Germany) using a T2^*^ sensitive gradient echo EPI sequence (TR = 2130 ms, TE = 40 ms, FA 90-, matrix size 64 x 64 x 32, voxel size 3.4 x 3.4 x 3.4 mm^3^). Thirty-two 3-mm thick transverse slices (FOV 22 x 22 cm^2^) were acquired, with a distance factor of 30%, covering the whole brain. Between 608 and 710 functional volumes were acquired for each session. The first three volumes were discarded to account for T1 saturation. A structural MR scan was obtained at the end of the session (T1-weighted 3D MP-RAGE sequence, TR = 1960 ms, TE = 4.4 ms, FOV 23 x 23 cm^2^, matrix size 256 x 256 x 176, voxel size 0.9 x 0.9 x 0.9 mm). Head movement was minimized by restraining the subject’s head using a vacuum cushion. Stimuli were displayed on a screen positioned at the rear of the scanner, which the participant could comfortably see through a mirror mounted on the standard head coil.

### fMRI data analyses

fMRI data were preprocessed using SPM5 (Wellcome Department of Imaging Neuroscience, http://www.fil.ion.ucl.ac.uk/spm) implemented in MATLAB version 7.0.4 (Mathworks Inc., Sherborn, MA). Functional scans were realigned using iterative rigid body transformations that minimize the residual sum of squares between the first and subsequent images. The scans were screened for motion artifacts and all time series with motion exceeding 3 mm (translation) or 3^°^ (rotation) were discarded. They were normalized to the MNI EPI template (voxel size: 2 X 2 X 2 mm) and spatially smoothed with a Gaussian kernel with full-width at half maximum (FWHM) of 8 mm (in order to minimize noise and to assure that the residual images conform to a lattice approximation of Gaussian random fields).

For each participant, BOLD responses were modeled at each voxel, using a general linear model with epoch regressors. Four conditions (decrease negative, watch negative, increase positive, and watch positive) were modeled as epoch-related responses. For each condition, each epoch ranged from the onset of the picture on the screen until the participant’s response. Boxcar functions representative of these epoch regressors were convolved with the canonical hemodynamic response. The design matrix also included the realignment parameters to account for any residual movement-related effect. A high pass filter was implemented using a cut-off period of 128 sec in order to remove the low-frequency drifts from the time series. Serial autocorrelations were estimated with a restricted maximum likehood algorithm with an autoregressive model of order 1 (+ white noise). Two contrasts looked for the differential main effects between the different conditions. We contrasted the positive regulation condition with the positive watch condition (positive regulation – positive watch) and the negative regulation condition with the negative watch condition (negative regulation – negative watch). The resulting set of voxel values constituted a map of *t* statistics [SPM {*T*}]. These summary statistics images were smoothed again (6-mm FWHM Gaussian kernel) in order to reduce remaining noise due to inter-subject differences in anatomical variability in the individual contrast images. They were then entered in a second-level analysis, corresponding to a random effects model, in order to account for inter-subject variance in each contrast of interest. Conjunction analyses assessed the commonality of activations in both groups (null conjunction; Friston, Penny, & Glaser, 2005). Two-sample *t* tests assessed group differences for the different contrasts. As a rule, statistical inferences were performed at the voxel level at *p* < 0.05 corrected (Family-Wise Error - FWE) for multiple comparisons across the entire brain volume, or small volume corrections at *p* < 0.05 for a priori locations of interest. This procedure was performed twice, once before the EC training and once after the training.

For a priori regions of interest, statistical inferences were corrected for multiple comparisons using Gaussian random field theory at the voxel level in a small spherical volume (radius 10 mm) around coordinates selected from the literature on emotion regulation and attention (Beauregard et al., 2001; Kim & Hamann, 2007; Ochsner et al., 2002, 2004). These a priori regions of interest concerned areas in the inferior frontal gyrus [± 56, 15, 14], the middle frontal gyrus [± 45, 12, 42], the superior frontal gyrus [± 21, 11, 48], the orbitofrontal gyrus [± 49, 31, -8], the inferior parietal gyrus [± 38, -64, 34], the inferior parietal lobule [± 60, -56, 42], the intraparietal sulcus [± 45, -43, 46], the anterior cingulate [± 8, 21, 28], and the amygdala [± 17, -8, -17].

## Results

### Behavioral Results

#### Self-ratings of emotional intelligence prior to the interventions

No baseline differences between the EC group and the improvisation group for emotional intelligence prior to the interventions were found, *t*(1, 34) = -0.15, *p* = .88 (Table 1).

**Table 1:** Means, standard deviations and significance of differences on emotional intelligence between EC and improvisation group prior and after interventions.

	EC group	Improvisation group	

Prior intervention	145.86 (20.23)	146.78 (16.79)	*t*(34) = -0.15, *p* = .879

After intervention	154.83 (23.25)	140.28 (0.05)	*t*(34) = 2.01, *p* = .05

#### Self-ratings of emotional arousal prior to the interventions

A 2 groups (EC vs. Improvisation) x 4 conditions (Decrease negative vs. Increase positive vs. Watch negative vs. Watch positive) repeated-measures analysis of variance (ANOVA) with on-line ratings for emotional arousal as the dependent variable was conducted. A significant main effect of condition was found, *F*(3, 102) = 81.47, *p* < .001. There were no baseline differences between the EC and the improvisation group on emotional arousal (Table 2). No significant interaction effect was found. Consistent with the predicted effect of regulation, participants reported greater arousal in the increase condition than the watch condition, *F*(1, 34) = 136.90, *p* < .001, and lower arousal in the decrease condition than the watch condition, *F*(1, 34) = 63.33, *p* < .001. To confirm that subjective arousal differed across stimulus types when participants were not actively attempting to regulate their emotional responses, an ANOVA was conducted with on-line arousal ratings for positive, negative, and neutral pictures that had been presented in the watch condition. Positive and negative pictures were rated higher than neutral pictures on arousal, *F*(1, 34) = 291.60, *p* < .001; *F*(1, 34) = 295.07, *p* < .001), respectively. Negative pictures were not rated higher than positive pictures on arousal, *F*(1, 34) = 2.86, *p* = .10.

**Table 2:** Means and standard deviations on-line ratings of emotional arousal during each condition and for each group before emotional competencies training.

	EC group	Improvisation group	

Conditions			

Decrease negative	2.00 (0.13)	1.93 (0.13)	*F*(1, 34) = 0.18, *p* = .68

Increase positive	3.20 (0.09)	3.19 (0.09)	*F*(1, 34) = 0.02, *p* = .89

Watch negative	2.56 (0.12)	2.53 (0.12)	*F*(1, 34) = 0.61, *p* = .44

Watch positive	2.52 (0.11)	2.39 (0.11)	*F*(1, 34) = 0.03, *p* = .87

#### Self-ratings of emotional competence after interventions

Results showed a significant main effect of group (*t*(1, 34) = 2.01, *p* = .05). The score of emotional competence was higher in the EC group than in the improvisation group (Table 1).

#### Self-ratings of emotional arousal after interventions

A 2 groups (EC vs. Control) x 4 conditions (Decrease negative, Increase positive, Watch negative and Watch positive) repeated-measures ANOVA with on-line ratings for emotional arousal as the dependent variable was conducted. As anticipated, analyses yielded a significant Group x Condition interaction, *F*(3, 102) = 5.61, *p* = .001, indicating a differential change on emotional arousal for the two groups and for specific conditions. For the decrease condition, the EC group reported lower arousal than the improvisation group, *F*(1, 34) = 4.07, *p* = .05. The EC group reported higher arousal than the improvisation group in the increase condition, *F*(1, 34) = 8.64, *p* < .01. The two groups showed no significant difference in the positive and negative watch conditions, *F*(1, 34) = 2.18, *p* < .15; *F*(1, 34) = 1.1, *p* = .30, respectively (Table 3). A significant main effect of condition was found, *F*(3, 102) = 66.26, < .001. The results are similar to those before emotional competencies training: Participants reported greater arousal during the increase condition than the watch condition, *F*(1, 34) = 95.9, *p* < .001, and lower arousal during the decrease condition than the watch condition, *F*(1, 34) = 72.72 *p* < .001. In the watch condition, positive and negative pictures were rated higher than neutral pictures on arousal, *F*(1, 34) = 211.14, *p* < .001; *F*(1, 34) = 346.62, *p* < .001, respectively. Negative pictures were not rated higher than positive pictures on arousal, *F*(1, 34) = 1.40, *p* = .25.

**Table 3:** Means and standard deviations on-line ratings of emotional arousal during each condition and for each group after emotional competencies training.

	EC group	Improvisation group	

Conditions			

Decrease negative	1.71 (0.12)	2.06 (0.12)	*F*(1, 34) = 4.07, *p* = .05

Increase positive	3.38 (0.09)	3.01 (0.09)	*F*(1, 34) = 8.64, *p* < .01

Watch negative	2.71 (0.11)	2.55 (0.11)	*F*(1, 34) = 1.11, *p* = .30

Watch positive	2.47 (0.12)	2.22 (0.12)	*F*(1, 34) = 2.18, *p* = .15

### Imaging Data

#### Prior to the EC intervention

**Figure 2:**
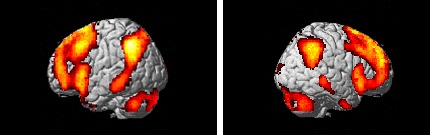
Activated brain regions for the contrast of decrease > watch for the negative pictures independently of the group before the EC training (first scanning). Two panels show left and right lateral views of regions active in the decrease negative – look negative contrast.

Brain regions associated with decreasing negative emotion were identified by comparing activations in the decrease negative condition and the watch negative condition (i.e., the decrease negative – watch negative contrast). The commonality of activations across the two groups was assessed via conjunction null analyses. For the decrease negative condition, both groups showed activation in a vast network of prefrontal areas including the bilateral inferior frontal gyrus, the bilateral middle frontal gyrus, the bilateral medial frontal gyrus, the bilateral superior frontal gyrus, and the left cingulate gyrus (Figure 2). In addition, the bilateral inferior parietal lobule, the bilateral supramarginal gyrus, the bilateral cerebellum, the right lingual gyrus, the bilateral middle temporal gyrus, and the left inferior temporal gyrus were also activated (Table 4).

**Table 4:** Brain regions associated with the down-regulation of negative pictures in both groups during the first scanning session before the EC training (p < .05, corrected for whole brain volume, if not otherwise specified). Note that the contrasts reflect activity relative to the watch negative condition. All coordinates refer to MNI voxel space.

Conjuction						

Anatomical region	Vox.	x	y	z	BA	Z-value

Decrease negative pictures > watch negative pictures						

Inferior frontal gyrus	470	-52	22	-4	47	5.06^*^

Inferior frontal gyrus	464	52	24	-10	47	5.47^*^

Inferior frontal gyrus	421	54	18	4	45	5.29^*^

Inferior frontal gyrus	297	-54	22	6	45	5.07^*^

Inferior frontal gyrus	156	-42	46	8	46	3.89^*^

Middle frontal gyrus	496	-40	16	44	8	6.27^*^

Middle frontal gyrus	370	46	26	40	9	4.66^*^

Middle frontal gyrus	283	-46	22	36	9	5.40^*^

Middle frontal gyrus	414	28	54	20	10	5.46^*^

Middle frontal gyrus	142	36	26	38	8	4.50^*^

Medial frontal gyrus	515	-6	28	44	8	6.09^*^

Medial frontal gyrus	515	2	38	48	8	5.69^*^

Superior frontal gyrus	433	-34	54	20	10	5.53^*^

Superior frontal gyrus	496	20	56	28	9	5.76^*^

Superior frontal gyrus	512	8	14	66	6	5.33^*^

Superior frontal gyrus	515	-12	12	69	6	5.39^*^

Anterior Cingulate gyrus	118	-8	32	32	32	4.35^*^

Inferior parietal lobule	506	-50	-50	46	40	6.69

Inferior parietal lobule	514	54	-50	50	40	6.02

Supramarginal gyrus	281	66	-52	30	40	5.70

Supramarginal gyrus	239	-60	-52	38	40	6.70

Cerebellum	505	-40	-56	-40	VIII	5.60

Cerebellum	470	46	-62	-38	CRII	5.07

Cerebellum	51	-4	-52	-22	V	3.47

Lingual gyrus	491	4	-90	-8	18	5.05

Middle Temporal gyrus	373	54	-30	-8	21	4.24

Middle Temporal gyrus	235	66	-34	-2	21	3.79

Inferior Temporal gyrus	279	-58	-34	-16	20	4.26

^*^significant at *p* < .05 after applying small volume corrections (see methods section for details).

Brain regions associated with increasing positive emotion were identified by comparing activations in the increase positive condition and the watch positive condition (i.e., the increase positive – watch positive contrast). The commonality of activations across the two groups was assessed via conjunction null analyses. There were no areas identified that exhibited significantly greater activation during the increase condition than the watch condition for positive pictures.

Next, we performed t-tests exploring group differences for the different regulation conditions. Analyses yielded no difference of cerebral activations between the two groups.

#### After EC training

**Figure 3:**
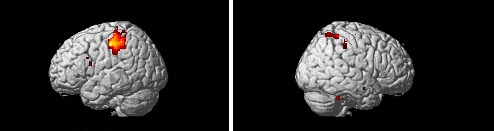
Regions more activated for the control group than the training group for the contrast of decrease > watch for the negative pictures after the EC training (second scanning). Two panels show left and right lateral views of regions more active in the decrease negative – watch negative contrast in the control group.

Group differences for the different regulation conditions after the EC training were assessed by t-tests. As shown in Table 5, the training group exhibited lower activation in the bilateral inferior parietal lobule, the right precentral gyrus, and the intraparietal sulcus as compared to the improvisation group for decrease negative condition (Figure 3).

**Table 5:** Maxima within regions showing BOLD signal changes in the decrease negative condition versus watch negative condition for training group versus improvisation group after the EC training (second scanning). Note that the contrasts reflect activity relative to the watch positive condition. All coordinates refer to MNI voxel space.

	Training group < Improvisation group						Improvisation group < Training group				

Anatomical region	Voxels	x	y	z	*BA*	SPM {Z}	Voxels	x	y	z	SPM {Z}

Inferior parietal lobule	422	-48	-30	50	40	4.12^*^	/				

Inferior parietal lobule	69	40	-32	44	40	3.39^*^	/				

Precentral gyrus	91	30	-32	54	4	3.22^*^	/				

Inferior parietal lobule / intraparietal sulcus	134	-32	-40	50		3.53^*^	/				

^*^significant at *p* < .05 after applying small volume corrections (see methods section for details).

**Figure 4:**
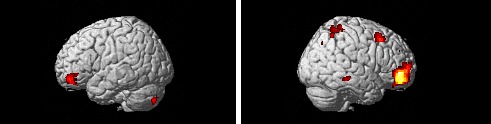
Regions more activated for the control group than the training group for the contrast of increase > watch for the positive pictures after the EC training (second scanning). Two panels show left and right lateral views of regions active in the increase positive – watch positive contrast in the control group.

As revealed in Table 6, the training group exhibited lower activation than the improvisation group in the right middle frontal gyrus, the left orbitofrontal gyrus and the right frontopolar cortex for the increase positive (Figure 4). However, no specific activation was found in the amygdala.

**Table 6:** Maxima within regions showing BOLD signal changes in the increase positive condition for training group versus control group after the EC training (second scanning). Note that the contrasts reflect activity relative to the watch positive condition. All coordinates refer to MNI voxel space.

	Training group < Improvisation group						Improvisation group < Training group				

Anatomical region	Voxels	x	y	z	*BA*	SPM {Z}	Voxels	x	y	z	SPM {Z}

Middle frontal gyrus	432	30	46	-2	10	5.06^*^	/				

Orbitofrontal gyrus	144	-28	46	-14	11	3.97^*^	/				

Middle frontal gyrus	223	44	18	48	8	3.75^*^	/				

Frontopolar cortex	402	32	54	-16	11	4.08^*^	/				

^*^significant at *p* < .05 after applying small volume corrections (see methods section for details).

## Discussion

To the best of our knowledge, this is the first imaging study examining the neural correlates of EC training. The main goal of the current study was to compare brain activity of individuals with and without emotional training in an emotional regulation task. We also investigated the neural correlates of emotion regulation for negative and positive emotional stimuli. More precisely, the inclusion of both increase positive and decrease negative regulation instructions allowed us to compare the neural correlates of the down-regulation of negative emotions and the up-regulation of positive emotions.

### Before EC training

On-line behavioral ratings of emotional arousal suggest that participants exploited successfully regulation strategies to modulate their subjective emotional reactions. Indeed, behavioral data showed that emotional regulation was effective during the emotional paradigm since participants significantly decreased negative emotion when intentionally down-regulating emotional responses and significantly increased positive emotion when intentionally up-regulating emotional responses.

Imaging results revealed activation in several prefrontal and cingulate regions implicated in emotion regulation and top-down cognitive control when participants were instructed to down-regulate their negative emotions. This result is consistent with prior findings examining the down-regulation of negative emotions displaying bilateral activation of prefrontal activation (Kim & Hamann, 2007; Ochsner et al., 2002; 2004; Phan et al., 2005). Decreasing negative emotions recruited the bilateral lateral PFC (LPFC, BA 9, 10, 45, 46), bilateral dorsomedial PFC (dmPFC, BA 6), bilateral medial PFC (MPFC, BA 9/10), bilateral lateral OFC (LOFC, BA 47), and left dorsal anterior cingulate (BA 32). The dorsal sector of the MPFC (i.e., superior frontal gyrus) has been implicated in maintaining spatial (Kim & Hamann, 2007; Petit, Courtney, Ungerleider, & Haxby, 1998) and non-spatial information during the delay for a response (Kim & Hamann, 2007; Petit et al., 1998). Recent findings suggested also that the dmPFC is involved during mentalizing (Spunt et al., 2011). Activation in this region during active regulation may reflect the maintenance of regulation strategies throughout each trial. The dorsal anterior cingulate has been implicated in the monitoring of ongoing responses (Botvinick, Braver, Barch, Carter, & Cohen, 2001; Kim & Hamann, 2007), suggesting that activity in this region may reflect the monitoring of internal and external emotional responses required for accurate feedback relevant to current regulatory goals. The OFC has been implicated in the down-regulation of negative emotions such as aggression and violence (Davidson et al., 2002; Davidson, Putnam, & Larson, 2000; Gur, Gunning-Dixon, Biker, & Gur, 2002; Kim & Hamann, 2007). The ventral part of the MPFC (BA 10), a region associated with self-referential processing and evaluation of internally generated information (Craik et al., 1999; D’argembeau et al., 2007; Kelly, Macrae, Wyland, Inati, & Heatherton, 2002) and perceived similarity between self and others was activated while decreasing negative emotions. This activation may reflect increased self-referential processing while the participants down-regulated negative emotions. Activity in the left lateral prefrontal area (BA 46) has been reported to be inversely related with the activity in the emotion-processing areas such as the amygdala and the medial OFC (Ochsner et al., 2002), suggesting that this region has a modulatory role for the down-regulation of emotion.

The activation of the cerebellum during emotional regulation found in the present study is in agreement with recent neuroimaging studies that have shown that the cerebellum could play a larger role than has previously been thought in complex cognitive processes, including the modulation of thought and emotion (Killgore & Yurgelun-Todd, 2007; Schmahmann, 2004; for a recent meta-analysis, Van Overwalle, Baetens, Mariën, & Vandekerckhove, 2014). In particular, the cerebellum is implicated in the regulation of emotion and mood, and some findings have reported cerebellum abnormalities in emotional disorders (Schutter & van Honk, 2009). The fact that the cerebellum is reciprocally connected to a broad range of limbic structures including the amygdala, hippocampus, and septum, as well as the prefrontal areas, provides a strong neuroanatomical argument in favor of cerebellum involvement in emotion regulation (Middleton & Strick, 2001; Snider & Maiti, 1976).

In sum, down-regulating negative emotion (1) activated regions of the lateral prefrontal cortex implicated in working memory and cognitive control (Knight, Staines, Swick, & Chao, 1999; Miller & Cohen, 2001, Ochsner et al., 2004; Smith & Jonides, 1999) that may support the generation and maintenance of regulation strategies, (2) activated the dorsal anterior cingulate, which is implicated in the on-line monitoring of performance (Botvinick et al., 2001; Ochsner et al., 2004; Ochsner & Feldmann Barrett, 2003), (3) activated regions of dorsal and ventral medial prefrontal cortex implicated in the self-monitoring and self-evaluation of emotion (Ochsner et al., 2004; Ochsner & Gross, 2004; Simpson et al., 2001), and (4) did not significantly modulate activation of the amygdala. .

The neural correlates of positive emotion regulation have remained largely unknown because few studies have examined this form of regulation (Beauregard et al., 2001; Kim & Hamann, 2007). Beauregard and colleagues (2001) showed that the attempt to inhibit the sexual arousal elicited by erotic film was associated with activation of the right superior frontal gyrus and the right anterior cingulate gyrus. Kim and Hamann (2007) found that positive emotion regulation engaged primarily left-lateralized prefrontal regions. In the present study, relative to the watch positive condition, up-regulation of positive stimuli was not associated with specific activation. No areas were identified that exhibited significantly greater activation during the increase condition than the watch condition for positive pictures. This pattern is not consistent with previous studies (Beauregard et al., 2001; Kim & Hamann, 2007) demonstrating that up-regulation showed an overlap of activations with the down-regulation of negative emotions, and also that several regions were uniquely activated during up-regulation of positive emotions such as the thalamus and caudate. One possible explanation is given by Schooler, Ariely, and Loewenstein (2003), who found that instructing people to try and feel as happy as they possibly could actually led to a decrease in momentary happy mood, relative to those who were not asked to try to be happy. It seems that happiness is very often the byproduct of an enjoyable experience, but perhaps it cannot be a deliberate goal in and of itself. These findings suggest that attempts to be happy can backfire. Both monitoring happiness and, more important, trying to be happy, produced a decline in happiness (Gross, 2006; Schooler et al., 2003). In addition, up-regulation of positive emotions can be influenced by cultural differences. For example, Verschuere, Crombez, and Koster (2001) found that affective ratings in a sample of Belgium students were less extreme compared to the North American ratings of the pictures from IAPS. Also, consistent with previous research, the association between valence and arousal is stronger for negative stimuli than for positive stimuli (Verschuere et al., 2001).

Prior studies have reported that amygdala activation is modulated by regulatory goals (Kim & Hamann, 2007; Oschner, 2002; 2004). However, in the present study, no amygdala activation was observed during the decrease negative or the increase positive conditions. Nevertheless, it has been shown that viewing complex unpleasant pictorial scenes elicits significantly weaker amygdala responses compared to viewing emotional facial expressions (Hariri, Tessitore, Mattay, Fera, & Weinberger, 2002; Nummenmaa, Hirvonen, Parkkola, & Hietanen, 2008). Moreover, we used relatively long blocks (10 seconds for picture presentation and 4 seconds to evaluate the intensity of affect). As the amygdala shows rapid habituation (Liberzon et al., 2000; Taylor et al., 1998), the long block duration may have attenuated the amygdala responses. Such kind of habituation has been observed during exposure to unpleasant visual stimuli (Liberzon et al., 2000; Taylor et al., 1998), fearful faces (Breiter et al., 1996; Phillips et al., 2001), novel ingroup faces (Hart et al., 2000), and complex visual stimuli (Fischer, Furmark, Wik, & Fredrikson, 2000). The temporal pattern of amygdala responses is more complex and dynamic than is captured by the temporally fixed (stationary) models that characterize most fMRI analyses.

### After emotional training

At behavioral level, the score on the TEIQue was higher in the EC group than in the improvisation group after the trainings. The EC group reported lower arousal than the improvisation group in the decrease condition and higher arousal than the improvisation group in the increase condition. These results suggest that emotion regulation is more effective in the training group than in the improvisation group.

Consistent to previous neuroimaging studies which suggested that individuals characterized by higher developed skills and abilities tend to show greater neural efficiency (Haslinger et al., 2004; Killgore & Yurgelun-Todd, 2007; Olson et al., 2006), the results of the present study show that participants in the training group exhibit lower activation than the improvisation group in the right middle frontal gyrus, the left orbitofrontal gyrus and the right frontopolar cortex when they increase their positive emotions. Prior studies have demonstrated that these PFC regions are major structures related to emotion regulation (Beauregard et al., 2001; Kim & Hamann, 2007; Ochsner et al., 2002; 2004). These PFC regions have been implicated in cognitive top-down control, strategy selection, implementation, and monitoring (MacDonald, Cohen, Stenger, & Carter, 2000; Ochsner & Gross, 2005). Lower activation found in this prefrontal cortical area among the training group is probably related to the expertise of participants, meaning that participants in the training group exhibited less effort to perform the task, resulting in lower activity in these regions.

Less cerebral activations among the training group as compared to the improvisation group were found in the bilateral inferior parietal lobule (BA 40), the right precentral gyrus (BA 4), and the intraparietal sulcus (BA 40) when they decreased negative emotion. This pattern of activation could reflect that the training group performed better on the task and with less attention than the improvisation group, as the inferior parietal lobule is a brain region implicated in attention (Culham, & Kanwisher, 2001; Majerus et al., 2010), working memory (Majerus et al., 2010; Rama, 2001), and in the processing of information related to self (Kircher et al., 2000). Activation in attentional regions suggests that the improvisation group may have generated greater effortful attention because the process of emotional regulation was less automatic than in the training group. The effect of practicing these emotion regulation tasks could be interpreted as an automaticity of performance, requiring less attentional resources.

In conclusion, the present study suggests that improvement of emotional competences produces a better emotional regulation, leading to greater expertise in regulating emotional stimuli, and inducing less activation within both PFC neural structures involved in top-down emotional regulation and inferior parietal lobules implicated in attention. Therefore, it could be argued that EC training induces a shift from explicit attention regulation processes (i.e., more effortful) to implicit ones (i.e., more automatic).

The present study has several limitations. First, since the study was conducted with female participants, our findings may not be applicable to men. We chose to include only women to avoid gender-related factors that might influence emotional responding (Bradley, Codispoti, Sabatinelli, & Lang, 2001; Wrase et al., 2003) or emotional regulation (McRae, Ochsner, Mauss, Gabrieli, & Gross, 2008; Rusting, 1998). Indeed, several findings showed that men and women differ in terms of brain activity associated with the appraisal of negative stimuli and during the voluntary control of emotional responses to aversive stimuli (Domes et al., 2010; McRae et al., 2008). Second, we only assessed one of the EC domains (i.e., emotional regulation). It would be interesting to replicate this study in evaluating more aspects of EC (e.g., utilization and understanding emotions). Third, we examined general negative and positive responses in a very simple context (picture viewing). In future research, it would be interesting to vary the complexity of the task and to examine a wider array of specific emotional responses (e.g., fear, sadness, contentment).

## Appendix: Outline of emotional competencies training sessions

### First Day

#### Session 1: Understanding emotions

- Welcome/Explanation of the sessions and introduction to the use of the personal diary.
- Introduction to the importance of emotions and explanation of key concepts (emotions, emotional competencies).
- Video clips illustrating the importance of emotions.
- Summary.

#### Session 2: Identifying emotions

- Review of previous session.
- Identifying one’s emotions using three doors (i.e., physiological activation, cognitions and action tendencies): theory and practice.
- Identifying other’s emotions through non verbal communication.
- Identifying other’s emotions through facial expression decoding: drill with the METT program.
- Summary and homework.

### Second day

#### Session 3: Listening other’s emotions

- Review of previous session and home­ work.
- Basic communication rules.
- Active listening.
- Empathic listening.
- Role play on active listening.
- Summary.

#### Session 4: Expressing emotions to others

- Review of previous session.
- How to express emotions: facts – emotions – needs – positive solutions.
- Role play on the expression of emotions.
- How to manage a conflict? Theory and Role play.
- Summary and homework.

### Third day

#### Session 5: Managing emotions

- Review of previous session and home work.
- Coping strategies and their effectiveness: theory and group discussion.
- Positive reappraisal: role play and drill.
- Mind-body connections and relaxation exercises.
- Summary.

#### Session 6: Enhancing positive emotions

- Review of previous session.
- The importance of positive emotions: theory and group discussion.
- Using the power of positive emotions: promoting positive feelings (e.g., gratefulness).
- Savouring: theory and exercises.
- Summary/Questions/Evaluation.
